# Patient satisfaction with HIV services in Vietnam: Status, service models and association with treatment outcome

**DOI:** 10.1371/journal.pone.0223723

**Published:** 2019-11-08

**Authors:** Bach Xuan Tran, Anh Kim Dang, Giang Thu Vu, Tung Thanh Tran, Carl A. Latkin, Cyrus S. H. Ho, Roger C. M. Ho

**Affiliations:** 1 Institute for Preventive Medicine and Public Health, Hanoi Medical University, Hanoi, Vietnam; 2 Bloomberg School of Public Health, Johns Hopkins University, Baltimore, Maryland, United States of America; 3 Institute for Global Health Innovations, Duy Tan University, Da Nang, Vietnam; 4 Center of Excellence in Evidence-based Medicine, Nguyen Tat Thanh University, Ho Chi Minh City, Vietnam; 5 Department of Psychological Medicine, National University Hospital, Singapore, Singapore; 6 Center of Excellence in Behavioral Medicine, Nguyen Tat Thanh University, Ho Chi Minh City, Vietnam; 7 Department of Psychological Medicine, Yong Loo Lin School of Medicine, National University of Singapore, Singapore, Singapore; 8 Institute for Health Innovation and Technology (iHealthtech), National University of Singapore, Singapore, Singapore; University of Ghana College of Health Sciences, GHANA

## Abstract

This study assessed the satisfaction of patients receiving antiretroviral treatment (ART) in Vietnam and its multilevel predictors. Cross-sectional data were collected from January to September 2013 in eight outpatient clinics in Hanoi and Nam Dinh provinces. Patient satisfaction was evaluated using the Satisfaction with HIV/AIDS Treatment Interview Scale. Multivariable Tobit regression was utilized to measure the associations between these factors and satisfaction with treatment services. Generalized Mixed-effect Regression model was used to estimate the effect of satisfaction with the quality of service on the change between the initial and the latest CD4 cell count. Among 1133 patients, most of them were completely satisfied with the 10 domains measured (65.5% to 82.5%). “Service quality and convenience” domain which was attributed by the waiting time and administrative procedure had the lowest score of complete satisfaction. Compared to central clinics, provincial clinics were negatively associated with the overall satisfaction (Coef = -0.58; 95%CI = -0.95; -0.21). Patients rating higher score in “Consultation, explanation, and guidance of health care workers”, “Responsiveness of health care workers to patients’ questions and requests” and “Perceived overall satisfaction with the quality of service” were related to improvement in immunological treatment outcomes. Our results revealed the high level of satisfaction among ART patients towards HIV care and treatment services, and this had a high correlation to treatment outcomes. Interventions should focus on reducing administrative procedures, providing sufficient guidance and comprehensive services which integrate physical with psychological care for improving the health outcome of the ART program.

## Introduction

Efforts to eliminate the global HIV/AIDS burden have achieved substantial progress in recent years [1], thanks to the advancement of interventions like antiretroviral therapy (ART). By 2017, 21.7 million among 31.6 million people living with HIV (PLWH) received ART, and the number of new infected HIV cases reduces by 47% in comparison with 1996 [2]. In Vietnam, since 2006, ART has been widely scaled with more than 120,000 PLWH having access to ART in 401 clinics at the end of 2017 [3]. This rapid expansion is critical; however, it raises a significant challenge to ensure the quality of services across clinics. Moreover, Vietnam has observed an accelerated shift from receiving foreign aids, which are the main sources of HIV response activities in Vietnam, to self-financing HIV/AIDS activities [4–6]. Therefore, optimizing HIV treatment and care services via regular monitoring and evaluating service quality is vital to ensure the sustainability of HIV programs in Vietnam.

In literature, the significant role of patient satisfaction indicators in evaluating the service quality has been well-documented [7–10]. Assessing patient satisfaction can also assist the estimation of service’s performance and the treatment outcomes as well as identification of patients’ unmet need [11]. Highly satisfied patients are more likely to build long-lasting relationships with health care providers, resulting in better treatment compliance and retention in care [12, 13]. In some chronic conditions such as colorectal cancer, patient satisfaction is found as an important predictor of survival [14]. Regarding HIV treatment, patient satisfaction links with ART adherence and retention [15]. Patients who are dissatisfied the with ART service quality have been reported to have an increased likelihood of developing drug-resistant strains [16].

In developing countries, patient satisfaction has received increasing attention in recent years [17–19]. A number of studies have been dedicated to figure out contextualized factors associated with satisfaction, including socio-economic and clinical characteristics, features of clinics and health system [20–23]. In Vietnam, however, little is known about this patient-reported outcome. To our knowledge, there was only one study conducted by Tran et al. which showed high satisfaction with service quality among methadone maintenance treatment (MMT) patients [11]. To fill the gap in the literature, we aimed to assess the satisfaction of patients receiving ART, the association between patient satisfaction and potential factors, as well as the HIV treatment outcome progress via CD4 cell count.

## Materials and methods

### Study design

A cross-sectional study were collected from January to September 2013 in eight outpatient clinics in Hanoi and Nam Dinh provinces. Hanoi and Nam Dinh are two provinces among those having the highest number of HIV-infected patients in the North of Vietnam [24]. In which, Hanoi remains a hotspot of illicit drug users while prostitution is more prevalent in Nam Dinh provinces. Vietnam health delivery model comprises four health administrative levels, including central, provincial, district, and commune-level (S1 Fig) [25]. Regarding HIV services delivery, there is the Department of Preventive Medicine and HIV / AIDS Prevention at the central level which under the management of the Ministry of Health, and provincial HIV / AIDS prevention centers in each province [26]. In addition, ART is recommended to be integrated into different types of services such as methadone maintenance treatment (MMT), HIV voluntary counseling and testing (VCT), general health care [27].

These clinics were chosen based on following inclusion criteria: 1) Offering ART services based on the official guideline of Vietnam Ministry of Health [28]; 2) Reflecting the diversity of health system (including central, provincial and district clinics) and 3) Covering both urban and rural areas. There were eight outpatient clinics selected in the study, including clinics at the central level (Bach Mai Hospital), provincial level (Nam Dinh provincial hospital and Nam Dinh Provincial AIDS Control Centre), and district level (Hoang Mai, Long Bien, Dong Anh, Ha Dong, Xuan Truong health centers).

The eligibility criteria for selecting participants were: 1) being available at one of the clinics within the study period; 2) aged 18 years old or more; 3) accepting to enroll in the study, and 4) having the capacity to answer interview from 15–20 minutes. Participants would be excluded from the study if they suffered from serious diseases during the interview time. We utilized a convenient sampling technique to recruit the respondents. The information of 1113 patients were entered into the analysis.

After agreeing to participate in the study, they were invited to a private room to assure their confidentiality. Then, interviewers introduced the objectives of this study and explained to patient’s that withdrawing from the interview would not affect the continuation and quality of their HIV care and treatment services. Finally, we collected written informed consents from patients who agreed to answer the survey. The study protocol was approved by the IRB of Vietnam Authority of HIV/AIDS Control.

### Measures and instruments

Patients interviews lasted 15 to 20 minutes and were conducted using the structured questionnaires by well-trained researchers of the Hanoi Medical University. The data collectors received a two-day intensive training session and participated in the pilot study with 20 patients in order to ensure the quality of data collected. The training session included the introduction of study purpose, data collection plan, and manners to communicate and interview HIV patients. None of the clinic staff was in involved in this procedure to avoid any social desirability bias.

We asked patients to report information on their socio-economic background (age, gender, education, marital status, and employment status). We also extracted clinical data regarding patients’ HIV stages and their ART treatment duration from their record books. HIV stages were determined based on the World Health Organization clinical staging of HIV/AIDS for adults and adolescents including stage I (asymptomatic), stage II (early HIV infection with several clinical manifestations), stage III (additional clinical manifestations), stage IV (AIDS) [29]. The number of CD4 cell count of patients were checked at the initial of the therapy and subsequently monitored every six months during the therapy. Researcher extracted initial and latest CD4 cell count based on results from medical records. Information about years from HIV diagnosis was self-reported. We also collected data about health status by using five questions from the Vietnamese version of the EuroQol-5 dimensions-5 levels. This instrument measured whether patients currently suffered from problems in mobility, self-care, usual activities, pain/discomfort and anxiety/ depression [30]. Self-reported adherence was evaluated by using a 100-point visual analogue scale, with 0 indicating “incomplete adherence” and 100 indicating “complete adherence” [31]. Optimal adherence were defined if adherence score were equal or higher than 95 points [31].

### Primary outcome

We evaluated patient satisfaction by employing the Satisfaction with HIV/AIDS Treatment Interview Scale (SATIS) Vietnamese version. This tool has been used successfully to assess patient satisfaction among HIV patients in previous studies in Vietnam [11, 32]. There were ten items in this instrument with a score ranging from 0 “complete dissatisfaction” to 10 “complete satisfaction.” These ten items can be categorized into three components including “Service Quality and Convenience”, “Availability and Responsiveness”, and “Competence of health care workers.” The score of each element is calculated by averaging the score of all items constructing this component. We computed the “Overall satisfaction” by averaging all item scores. The scores of three components and “Overall satisfaction” are from 0 to 10, which higher score means higher satisfaction [11, 32]. We also evaluated perceived overall satisfaction with the quality of service and the effectiveness of service by using a five-point Likert scale (from “completely satisfied” (level 5) to “completely dissatisfied” (level 1).

### Statistical analysis

Data were analyzed using STATA version 12 (Stata Corp. LP, College Station, United States of America). One-way ANOVA and Chi-square test were used to examine the differences in socioeconomic characteristics, clinical factors, and patient’s satisfaction among clinics in different level of health systems. Multivariable tobit regression was employed to examine the associations between three components of satisfaction and overall satisfaction with related factors. Generalized mixed-effect model, with gaussian family and identity link, was used to estimate the effect of each item of SATIS instrument as well as overall satisfaction on the change of initial and the latest CD4 cell count. A value of p <0.05 was considered the statistical significance.

### Ethics approval

The study protocol was approved by the IRB of Vietnam Authority of HIV/AIDS Control’s Scientific Research Committee (28/QD-AIDS). All respondents were asked to provide written informed consents. They could withdraw at any time without any influences on their current treatment.

## Results

Among 1133 patients, the majority were males (58.7%), 57.3% had less than high school education, 61.2% lived with spouse/partners, and 41.4% were self-employed. The mean age was 35.5 (SD = 6.9) years old. Most of participants received treatment in Hanoi (88.4%), health center/AIDS center (51.5%), urban areas (77.2%) and district clinics (51.4%) (Table 1).

**Table 1:** Demographic characteristics of respondents and facilities (n = 1133).

Characteristics	n	%
**Gender (Male)**	665	58.7
**Education attainment**		
< High school	649	57.3
High school	362	32.0
> High school	121	10.7
**Marital status**		
Single	169	14.9
Live with spouse/partners	693	61.2
Separate/Divorce/Widow	271	23.9
**Occupation**		
Unemployed	232	20.5
Self-employed	469	41.4
White Collar	80	7.1
Workers/Farmers	282	24.9
Others	70	6.2
**Setting**		
Nam Dinh	132	11.7
Hanoi	1001	88.4
**Type of facility**		
Hospital	549	48.5
Health center HIV/AIDS center	584	51.5
**Location**		
Urban	875	77.2
Rural	258	22.8
**Level of health system**		
Central	277	24.5
Provincial	273	24.1
District	583	51.4
	**Mean**	**SD**
**Age**	35.5	6.9

About 42% of the respondents were at stage I of HIV (Table 2). The percentage of respondents in provincial clinics having any problems in five health conditions were significantly higher than those in other clinics (p<0.05). The mean initial CD4 cell count was 120.7 (SD = 141.8) cells/μL. Most of the participants had the number of current CD4 cell count under 200 cells/μL.

**Table 2:** Clinical characteristics and treatment outcomes among respondents (n = 1133).

Characteristics	Central clinic (n = 277)		Provincial clinic (n = 273)		District clinic (n = 583)		Total		p-value
	n	%	n	%	n	%	n	%	
**HIV stage**									
Stage I	85	31.6	183	69.9	188	34.1	456	42.1	<0.01*
Stage II and III	44	16.4	39	14.9	110	20.0	193	17.8	
Stage IV (AIDS)	27	10.0	16	6.1	58	10.5	101	9.3	
Unknown	113	42.0	24	9.2	195	35.4	332	30.7	
**Having problems in**									
Mobility	30	10.8	81	29.7	121	20.8	232	20.5	<0.01*
Self-care	21	7.6	47	17.2	42	7.2	110	9.7	<0.01*
Usual activities	30	10.8	67	24.5	91	15.6	188	16.6	<0.01*
Pain/Discomfort	69	24.9	146	53.5	212	36.4	427	37.7	<0.01*
Anxiety/Depression	82	29.6	177	64.8	250	42.9	509	44.9	<0.01*
**Number of current CD4 cell (cells/μL)**									
<200	195	70.4	208	76.2	399	68.4	802	70.8	0.24*
≥200 and <350	55	19.9	40	14.7	130	22.3	225	19.9	
≥350 and <500	9	3.3	9	3.3	23	4.0	41	3.6	
≥500	18	6.5	16	5.9	31	5.3	65	5.7	
**Optimal adherence to ART**	203	73.3	203	74.4	414	71.0	820	72.4	0.55*
	**Mean**	**SD**	**Mean**	**SD**	**Mean**	**SD**	**Mean**	**SD**	
**Initial CD4 cell count** (cells/μL)	113.9	133.6	103.3	162.3	132.2	134.1	120.7	141.8	<0.01^#^
**Years since diagnosing HIV** (+)	3.8	3.6	5.0	3.3	5.6	3.2	5.0	3.4	<0.01^#^
**Duration of ART (years)**	3.3	2.0	4.6	2.0	4.8	2.2	4.4	2.2	<0.01^#^
**VAS Adherence**	93.8	12.7	93.6	11.3	94.5	8.8	94.1	10.4	0.73^#^

*Chi square test,

^#^One-way Anova test

Patients’ satisfaction measured by SATIS is described in Table 3. Most of the respondents were completely satisfied with the ten domains measured (65.5% to 82.5%). The highest average score was in “Competence of health workers” (9.6±1.1). The proportion of patients being completely satisfied with all items as well as the average score of each domain in central clinics were significantly higher than in other clinics (p<0.05).

**Table 3:** Patients’ satisfaction measured by Satisfaction with HIV/AIDS Treatment Interview Scale (SATIS) (n = 1133).

Characteristics	Central clinic (n = 277)		Provincial clinic (n = 273)		District clinic (n = 583)		Total		p-value
	n	%	n	%	n	%	n	%	
**% Completely satisfied with (SATIS instrument)**									
Quality of services delivery	207	74.7	155	56.8	380	65.2	742	65.5	<0.01*
Access to information and guidance on hospital services and procedures	216	78.0	169	61.9	398	68.3	783	69.1	<0.01*
Consultation, explanation, and guidance of health care workers	225	81.2	184	67.4	414	71.0	823	72.6	<0.01*
Convenience in check-up booking, waiting time, and administrative procedure	212	76.5	174	63.7	363	62.3	749	66.1	<0.01*
Convenience in using related medical services within the facility, e.g. lab tests, referrals, or related examinations by different specialists	217	78.3	153	56.0	387	66.4	757	66.8	<0.01*
Inter-professional and inter-departmental collaborations	218	78.7	147	53.9	394	67.6	759	67.0	<0.01*
Competence of health care workers	226	81.6	178	65.2	405	69.5	809	71.4	<0.01*
Responsiveness of health care workers to patients’ questions and requests	230	83.0	201	73.6	421	72.2	852	75.2	<0.01*
Availability of patients’ needed health care services	225	81.2	190	69.6	401	68.8	816	72.0	<0.01*
Medical confidentiality and respect of patients’ privacy	248	89.5	224	82.1	463	79.4	935	82.5	<0.01*
	**Mean**	**SD**	**Mean**	**SD**	**Mean**	**SD**	**Mean**	**SD**	
**SATIS domains**									
Service quality and convenience	9.6	1.1	9.1	1.4	9.4	1.2	9.4	1.2	<0.01^#^
Availability and responsiveness	9.6	1.1	9.3	1.4	9.3	1.3	9.4	1.2	<0.01^#^
Competence of health workers	9.7	1.2	9.5	1.3	9.5	1.2	9.6	1.1	0.04^#^
Overall satisfaction	9.6	1.1	9.2	1.3	9.4	1.1	9.4	1.1	<0.01^#^
**General perceived satisfaction**									
Perceived overall satisfaction with the quality of service	4.6	0.5	4.5	0.7	4.5	0.6	4.5	0.6	0.06^#^
Perceived overall satisfaction with the effectiveness of service	4.6	0.5	4.5	0.7	4.5	0.6	4.5	0.6	0.18^#^

*Chi square test,

^#^One-way Anova test

Table 4 indicates that patients with a higher level of education had lower satisfaction with “Service quality and convenience” and “Overall satisfaction”. Likewise, patients at stage II, III and IV (AIDS), and having problems of pain/discomfort had lower satisfaction with all domains and overall satisfaction compared to the reference groups (patients at stage I and those without pain/discomfort problems, respectively).

**Table 4:** Associated factors with the patient satisfaction.

Characteristics	Service quality and convenience		Availability and responsiveness		Competence of health workers		Overall satisfaction	
	Coef.	95%CI	Coef.	95%CI	Coef.	95%CI	Coef.	95%CI
**Individual characteristics**								
**Age** (year)	0.01	-0.01; 0.03	0.01	-0.01; 0.04	0.02*	-0.00; 0.05	0.01*	-0.00; 0.03
**Gender** (Female vs Male)	0.23*	-0.04; 0.49	0.29*	-0.01; 0.59	0.32*	-0.06; 0.69	0.21*	-0.02; 0.45
**HIV Stage** (vs stage I)								
Stage II and III	-0.3	-0.65; 0.06	-0.45*	-0.85; -0.04	-0.48*	-0.98; 0.01	-0.32*	-0.64; -0.00
Stage IV (AIDS)	-0.50*	-0.94; -0.07	-0.64*	-1.13; -0.14	-0.68*	-1.29; -0.07	-0.46*	-0.85; -0.07
Unknown	0	-0.32; 0.33	-0.01	-0.39; 0.36	0	-0.47; 0.46	0.01	-0.28; 0.31
**Duration of ART (year)**	-0.04	-0.10; 0.02	-0.03	-0.10; 0.03	-0.07*	-0.16; 0.01	-0.04	-0.09; 0.02
**Having problems in mobility** (Yes vs No)	-0.16	-0.59; 0.26	-0.14	-0.62; 0.35	-0.54*	-1.12; 0.04	-0.18	-0.56; 0.20
**Having problems in self-care** (Yes vs No)	0.45	-0.10; 1.00	0.32	-0.31; 0.95	0.5	-0.27; 1.27	0.38	-0.11; 0.88
**Having problems in usual activity** (Yes vs No)	-0.17	-0.66; 0.31	-0.33	-0.88; 0.23	0.17	-0.52; 0.86	-0.18	-0.61; 0.26
**Pain/ Discomfort** (Yes vs No)	-0.65*	-0.97; -0.33	-0.61*	-0.98; -0.25	-0.51*	-0.97; -0.06	-0.51*	-0.80; -0.22
**Anxiety/Depression** (Yes vs No)	0.07	-0.23; 0.38	0.17	-0.18; 0.52	0.05	-0.39; 0.48	0.05	-0.22; 0.33
**Facility factors**								
**Level of health system** (vs Central)								
Provincial	-0.66*	-1.07; -0.24	-0.92*	-1.41; -0.44	-0.71*	-1.31; -0.11	-0.58*	-0.95; -0.21
District	-9.74	-599.41; 579.93	-10.15	-505.37; 485.07	-10.71	-440.65; 419.24	-8.49	-318.36; 301.37
**Type of facility** (Health center vs Hospital)	9.21	-580.46; 598.88	9.34	-485.89; 504.56	10.04	-419.91; 439.98	7.94	-301.93; 317.80
**Province** (Hanoi vs Nam Dinh)	1.42*	1.02; 1.82	1.24*	0.78; 1.69	1.56*	1.02; 2.11	1.29*	0.93; 1.65
**Location** (Rural vs Urban)	0.40*	0.03; 0.76	0.42*	0.00; 0.84	0.11	-0.40; 0.62	0.41*	0.08; 0.73

*p-value <0.05

Patients receiving treatment in provincial clinics had lower satisfaction in all domains than those in central clinics. Otherwise, patients enrolled in clinics in Hanoi had higher satisfaction than those in Nam Dinh province. Receiving treatment in rural clinics correlated with higher satisfaction in “Service quality and convenience”, “Availability and responsiveness”, and overall satisfaction.

After adjusting for socio-economic and clinical characteristics, Table 5 revealed that patients who had a higher score in “Access to information and guidance on hospital services and procedures” reduced their latest CD4 cell count compared to baseline CD4 cell count (Coef. = -27.85, p<0.05). Meanwhile, people rating higher score in “Consultation, explanation, and guidance of health care workers”, “Responsiveness of health care workers to patients’ questions and requests” and “Perceived overall satisfaction with the quality of service” had increased CD4 cell count in the most recent test compared to the baseline CD4 cell count.

**Table 5:** Associations between patient satisfaction and change of initial and the latest CD4 cell count.

Characteristics	Change of initial and the latest CD4 cell count		
	Coef.	95%CI	p-value
**Age (year)**	-1.41	-2.64; -0.18	0.03
**Gender** (Female vs Male)	28.03	11.14; 44.91	0.01
**Level of education**	5.11	-2.42; 12.64	0.18
**HIV Stage (vs Asymptomatic)**			
Symptomatic	-39.02	-62.08; -15.96	0.01
AIDS	-46.07	-75.11; -17.03	0.01
Unknown	-27.75	-46.95; -8.55	0.01
**Having problems in mobility** (Yes vs No)	-17.13	-45.29; 11.03	0.23
**Having problems in self-care** (Yes vs No)	-38.44	-75.77; -1.11	0.04
**Having problems in usual activity** (Yes vs No)	7.17	-25.32; 39.66	0.67
**Pain/ Discomfort** (Yes vs No)	-12.33	-33.49; 8.83	0.25
**Anxiety/ Depression** (Yes vs No)	-26.89	-46.4; -7.37	0.01
**Duration of ART (year)**	11.81	8.04; 15.08	0.01
**Adherence VAS score**	0.39	-0.4; 1.18	0.33
**SATIS items (10-point scale)**			
Quality of services delivery (Yes vs No)	3.83	-7.28; 14.95	0.5
Access to information and guidance on hospital services and procedures (Yes vs No)	-27.85	-45.48; -10.23	0.01
Consultation, explanation, and guidance of health care workers (Yes vs No)	31.6	13.83; 49.37	0.01
Convenience in check-up booking, waiting time, and administrative procedure (Yes vs No)	-5.15	-18.71; 8.42	0.46
Convenience in using related medical services within the facility, e.g. lab tests, referrals, or related examinations by different specialists (Yes vs No)	-13.38	-29.85; 3.09	0.11
Inter-professional and inter-departmental collaborations (Yes vs No)	7.27	-4.78; 19.32	0.24
Competence of health care workers (Yes vs No)	-3.42	-21.46; 14.62	0.71
Responsiveness of health care workers to patients’ questions and requests (Yes vs No)	32.53	14.41; 50.65	0.01
Availability of patients’ needed health care services (Yes vs No)	-12.19	-27.24; 2.86	0.11
Medical confidentiality and respect of patients’ privacy (Yes vs No)	-10.07	-23.783.64	0.15
**Overall satisfaction**			
Perceived overall satisfaction with the quality of service (5-point scale)	34.46	5.24; 63.69	0.02
Perceived overall satisfaction with the effectiveness of service (5-point scale)	-27.37	-55.22; 0.48	0.05

## Discussion

Our study attempted to set some light on the satisfaction of patients receiving ART in Vietnam. Using SATIS instrument, a high proportion of patients was found to be satisfied with three sub-domains “Service quality and convenience”, “Availability and responsiveness” and “Competence of health workers”, suggesting the acceptance for quality of services. Higher level of satisfaction with the capacity of medical staff in communicating with patients was positively associated with higher number of CD4 cell count.

The improvement of quality of ART services plays a vital role since ART services were recommended to initiate as soon as possible, and these services have rapidly scaled up [33]. Overall, all three sub-domains were highly evaluated which is consistent with a previous study [11]. The score of competence of health staff domain was observed to be higher than the score of service quality as well as availability and responsiveness. High satisfaction with the competency of doctors can be explained by precise diagnosis and adequate explanation of health problems for patients to enhance their health status greatly [34]. "Service quality and convenience" domain which was attributed by the waiting time and administrative procedure had the lowest score of complete satisfaction, which is similar to other studies [35, 36]. Long waiting time and complicated administrative procedure should be considered as barriers for ART patients when accessing and utilizing HIV care and treatment services. Moreover, findings from our study suggested that HIV/AIDS infected patients received deficient information for their treatment process from health workers. This result contrasts with the previous study which indicated sufficient competency of nurses in providing HIV/AIDS information as well as being appropriately responsive [37].

When investigating the relationship between patients’ satisfaction and treatment outcomes, we found that higher perceived overall satisfaction with the quality of service was positively associated with the change of the number of CD4 cell count. Previous studies revealed that patient satisfaction with healthcare services was related to retention in and adherence to ART treatment, which acts as a critical factor of HIV suppression [15, 38]. Our result suggests that patient-centered interventions where satisfaction with treatment services is enhanced can be effective in optimizing HIV outcomes. Specifically, the higher satisfaction with the competency of health care workers (consultation or explanation; responsiveness to patients’ requests) was related to reducing HIV virus replication. Those who were dissatisfied with the relationship between them and their physician tended to miss more ART doses compared to those who were satisfied [38]. Moreover, having medical checkup regularly was better if doctors carefully consult and explain their health status as well as promptly respond to their requests [39]. Therefore, our study’s result contextualized evidence to inform aspects should be enhanced, which might lead to CD4 improvement.

Noticeably, we found that patients who received ART treatment in provincial hospitals were less likely to satisfy with HIV care and treatment services in all dimensions. Patients usually expect that national hospitals will provide better care and treatment services which base on larger intake capacity, better techniques and famous doctors [40, 41]. In Vietnam, it has been reported that, while 48% of patients visit national hospitals, only 18% of them come to the correct level of health services [42]. Along with the above reasons, because the majority of national hospitals located at Hanoi, patients treated at health facilities in Hanoi were more likely to completely satisfy with HIV treatment services compared to those treated Nam Dinh. Moreover, participants who suffered from pain and discomfort and who were at stage IV (AIDS) had a lower score of satisfaction in three domains and overall quality of services and treatment outcome. The result may suggest that ART patients would more prefer for comprehensive care clinics, which integrate physical and psychological care rather than only ART treatment services [32]. In addition, at the stage of AIDS, patients have to undergo many opportunistic infections and less effective in treatment results. Therefore they may expect a better quality of health care services [43, 44].

This is one of the very first studies assessing patients’ satisfaction regarding ART treatment services in a diverse level of healthcare delivery model in Vietnam. Findings from our study have an important contribution to the expansion of ART services as higher satisfaction were strongly associated with better HIV treatment outcomes. Our study suggests several implications. First, the administrative procedures should be shortened and followed the guidelines which may reduce the inconvenience for ART patients. Second, to enhance the level of satisfaction in provincial hospitals, the capacity of health workers, a better quality of treatment services and sufficient guidance for ART patients should be under consideration. Third, integrating physical with psychological care is critical as ART patients prefer for comprehensive care clinics instead of ART treatment alone. Finally, the information regarding satisfaction of ART patients should be collected regularly because determining the flaw of health services as well as enhancing the quality of these services should be based on expectations and the feedback of patients.

The strengths of this study are utilizing the validated instruments to assess patient satisfaction towards ART treatment and quality of services. This study also conducted on a large pool of ART patients in various clinics with multiple levels of the health system (national, provincial and district center). However, several limitations should be acknowledged. Convenient sampling technique may limit the ability to generalize the findings to the whole population. Second, causal relations between patients’ satisfaction and health outcome cannot be established due to the cross-sectional design. Third, we collected data based on self-reporting which may cause recall bias. Social desirability bias should be acknowledged as patients were asked to assess health facility which they received treatment. To handle social desirability bias, patients were invited into a private counseling room and be interviewed by researchers who were not staff of the hospital. All answers and personal information of patients were secured and only used for study purpose. Finally, since only data of the initial and latest CD4 cell count were examined, data of number CD4 cell count during the therapy were not reported. A further longitudinal study in the future is necessary to address these limitations and improve the planning of ART program in Vietnam.

## Conclusions

In conclusion, our results revealed the high level of satisfaction among ART patients towards HIV care and treatment services and this had a high correlation to treatment outcomes. However, results were different across service models. Interventions should focus on reducing administrative procedures, providing sufficient guidance and comprehensive services which integrate physical with psychological care for improving the health outcome of the ART program.

## Supporting information

S1 Fig Outline of Vietnamese health delivery models. (PDF)
