# Supplementary material for: Patient satisfaction with HIV services in Vietnam: Status, service models and association with treatment outcome
Source: PLoS One. 2019 Nov 8;14(11):e0223723. doi: 10.1371/journal.pone.0223723 (PMC6839840; doi:10.1371/journal.pone.0223723)
Supplement: S1 Fig — (PDF) [file pone.0223723.s001.pdf]

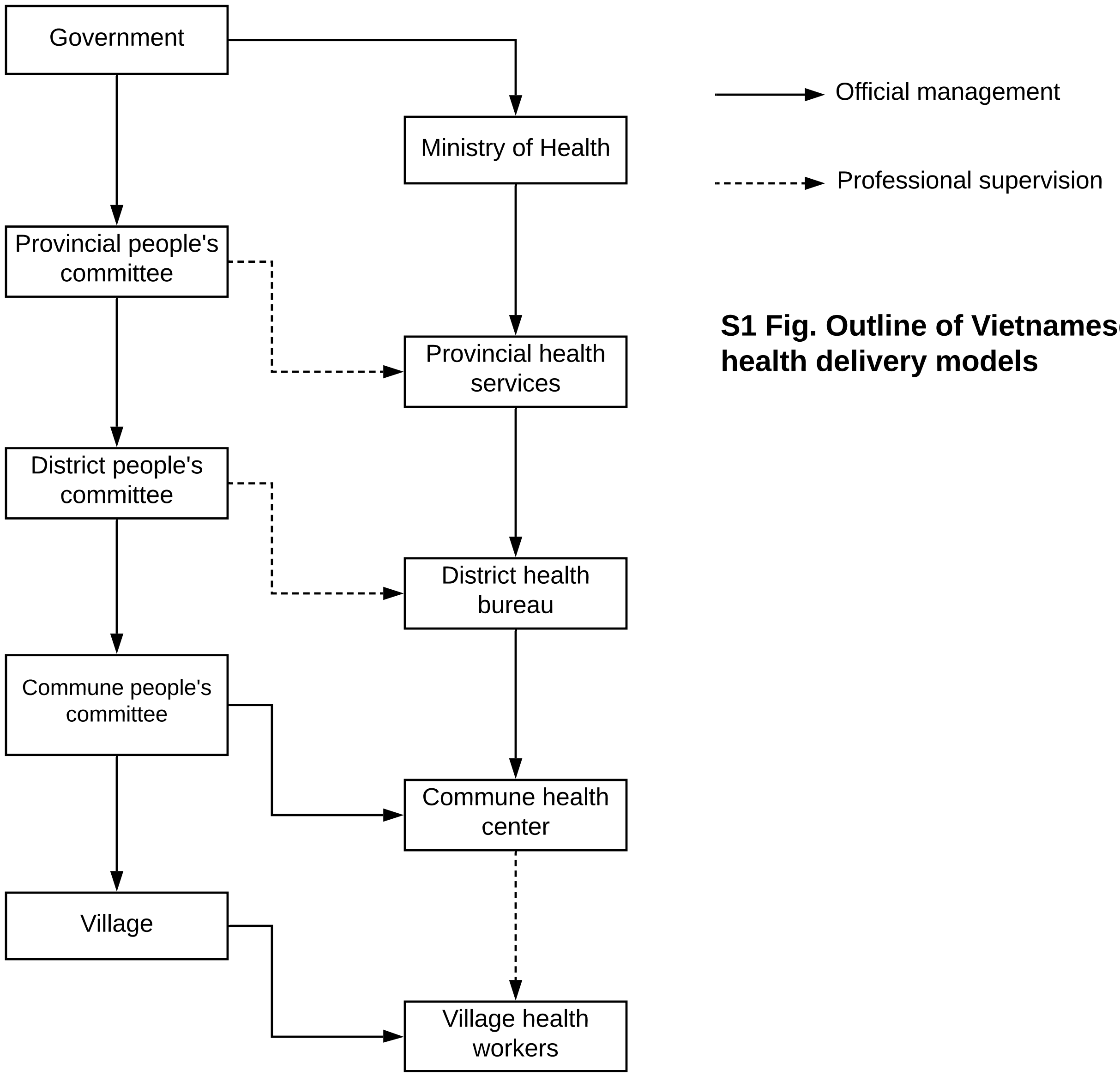

S1 Fig. Outline of Vietnamese health delivery models
